# Motivations to eat healthily in older Dutch adults – a cross sectional study

**DOI:** 10.1186/s12966-014-0141-9

**Published:** 2014-11-18

**Authors:** S Coosje Dijkstra, Judith E Neter, Ingeborg A Brouwer, Martijn Huisman, Marjolein Visser

**Affiliations:** Department of Health Sciences and the EMGO Institute for Health and Care Research, Faculty of Earth and Life Sciences, VU University Amsterdam, De Boelelaan 1085, 1081 HV Amsterdam, the Netherlands; Department of Epidemiology and Biostatistics, VU University Medical Center, Amsterdam, the Netherlands; Department of Sociology, VU University Amsterdam, Amsterdam, the Netherlands; Department of Psychiatry, VU University Medical Center, Amsterdam, the Netherlands

**Keywords:** Motivations, Older adults, Healthy eating, Socio-economic position

## Abstract

**Background:**

To influence dietary behaviors, more insight in food choice motivations is necessary. This study identified what motivations older adults have to eat healthily and investigated to what extent these motivations are particular to specific subgroups according to socio-economic position and other demographic, lifestyle and health characteristics.

**Methods:**

We used data from 1,050 older Dutch adults who participated in the Longitudinal Aging Study Amsterdam (65-80 years, independently living, normal cognitive status). Motivations to eat healthily and characteristics were measured with a self-reported questionnaire. We used logistic regression analyses to estimate odds ratios (OR) and 95% CI for the association between subgroups and motivations to eat healthily.

**Results:**

The most reported motivations to eat healthily were: “feeling fit” (51.7%), “current health” (49.7%) and “body weight” (39.2%). Multivariate analyses showed that older adults with chronic diseases (≥2 vs. no chronic disease OR: 4.41, 95% CI: 2.31-8.44) and a poor self-rated health (poor vs. good OR: 2.31, 95% CI: 1.22-3.73) were more likely to report “current disease” as a motivation to eat healthily. Groups from lower socio-economic positions were less likely to report “to prevent diseases” (low income vs. high OR: 0.52, 95% CI: 0.32-0.86, low education vs. high OR: 0.43, 95% CI: 0.27-0.70) and older adults with obesity were less likely to report “current health” (obese vs. normal weight OR: 0.47, 95% CI: 0.32-0.69) as motivations to eat healthily.

**Conclusion:**

Multivariate analyses showed that the presence of a disease in older adults is an important motivation for them to eat healthily, which might indicate that older adults with health problems are aware of the link between their disease and nutrition. Older adults from lower socio-economic positions or those with obesity require a specific approach because disease prevention seems to be of lesser importance for these groups, even though a healthy diet could improve their health. Future research should investigate the reasons behind the motives of low socio-economic position and obese older adults.

## Background

Western populations are aging and this will have significant social and economic consequences. In the Netherlands, 17% of the population is aged 65 years and older [1]. This is around the European average, but higher than the proportion in the United States (13%) [2]. Nowadays, an average Dutch person aged 65 years is expected to live another 20 years [3]. Life expectancy continues to increase throughout the world. It is estimated that in 2040, 26% of the population will be aged 65 or older [2]. This will lead to a greater awareness of the importance of improving the quality of life of older adults by all parties involved.

Studies examining lifestyle characteristics in relation to health among older adults showed that nutrition is an important modifiable determinant of health and that a healthy diet can add years to life, as well as improve the quality of life in old age [4-7]. Despite continuous efforts to promote a healthy diet, the dietary intake of older adults still does not meet the recommendations [8-10].

Healthy food choices are influenced by a complex combination of many factors. Studies in younger adults showed that health motivations and expectations are of major importance to people’s food choice [11,12]. The later adulthood is a critical period for healthy eating as chronic diseases will typically present themselves during this stage in life and the consequences of dietary change may be noticed more directly. However, few studies have investigated the underlying motivations for older adults to eat healthily. This information provides insight in why older adults want to eat healthily and is important for the development of effective interventions aimed at improving the dietary quality and subsequently the health of older adults. Two qualitative studies showed that the desire to stay healthy, to regain or maintain fitness and to preserve general health positively influenced food choice in older adults [2,13]. Others showed that the desire to improve the quality of life, to increase longevity, and to prevent diseases were strong motivating factors for eating healthily in older adults [14].

The older population in particular is a heterogeneous group and behaviors may differ by subgroup, i.e. socio-economic position (SEP) or health status. Therefore, it is not only important to know what motivates older adults to eat healthily, but also to identify potential differences in motivations between these subgroups. It has been suggested that motivations for healthy eating may play a role in explaining SEP differences in diet [12]. A previous study in older adults showed that those with higher education levels were more likely to report the prevention of disease as a motivation for healthy eating [14], suggesting that they acknowledge the link between healthy eating and health. Others showed that adults with lower incomes place more importance on price than health in their food choice compared to those with higher incomes [15,16], but very few studies have tested this in older adults.

The aims of the current study were first to identify what motivations older people have to eat healthily in a large population-based sample and second, to investigate to what extent these motivations are particular to specific subgroups according to SEP and other demographic, lifestyle and health characteristics.

## Methods

### Respondents

We used data from the Longitudinal Aging Study Amsterdam (LASA), an ongoing cohort study originally designed to study the determinants, trajectories and consequences of physical, cognitive, emotional and social functioning in relation to aging in the Netherlands. Details on the sampling and data collection procedures have been described elsewhere [17]. In summary, a random sample of older individuals aged 55-85 years, stratified by age, sex, level of urbanization and expected five year mortality, was drawn from the population registers of eleven municipalities in three geographical areas in the Netherlands. In total, 3,107 subjects who were representative of the Dutch older population were enrolled in the baseline examination (1992-1993). In 2002-2003, a new cohort of 1,002 subjects, aged 55-65 years was added to the study using the same sampling procedures. Follow up examinations were repeated every three years.

The source population for the current study consisted of 2,165 LASA respondents who participated in the fourth LASA cycle (2005-2006) and were invited to participate in the LASA Lifestyle Study, an ancillary cross sectional study that was conducted in 2007. Eligibility criteria were: age <80 years, independently living and having a Mini Mental State Examination score >23, by which we attempt to exclude older adults with poor cognitive status. In total 1,421 respondents met these criteria, of whom 1,058 completed a self-administered lifestyle questionnaire by mail (response rate 74.5%, 326 no response, 18 refused, 8 were not able due to physical problems and 11 deceased). We excluded 8 persons for whom all data on motivations for healthy eating were missing (N = 1,050).

This study was conducted according to the guidelines laid down in the Declaration of Helsinki and was approved by the ethical review board of the VU University Medical Center (Amsterdam, the Netherlands). All participants gave written informed consent.

### Motivations

**Figure 1:**
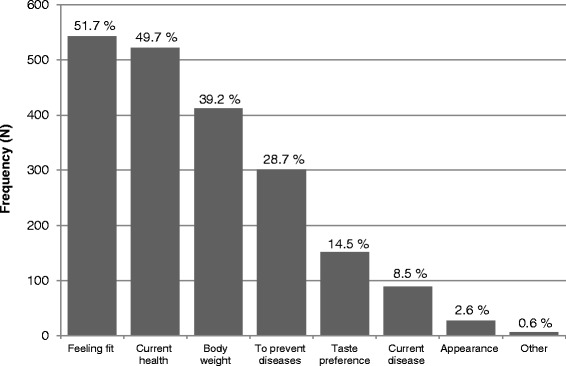
**Prevalence of reported motivations to eat healthily in older adults.**

We assessed the motivations for healthy eating by asking respondents “Which of the following factors are most important for you to eat healthily?” They could choose a maximum of two responses from a list of seven motivations (Figure 1). These motivations were based on the Food Choice Questionnaire [18,19]. This questionnaire is designed to measure motivations underlying the selection of foods. We selected those motivations that were applicable for older adults. They could also choose the option “Other, being….” and fill out their most important motivation to eat healthily.

### Potential factors associated with motivations to eat healthily

Potential factors associated with motivation to eat healthily included: age, gender, body mass index (BMI), smoking, alcohol use, SEP, the number of diet-related chronic diseases, depressive symptoms, self-rated health, walking disability and nutritional knowledge [9,14,20]. We calculated BMI as measured weight in kilograms divided by measured height in meters squared. Weight status was categorized as underweight: BMI <18.5 kg/m^2^, normal: BMI 18.5-24.9 kg/m^2^, overweight: BMI 25-29.9 kg/m^2^ or obesity: BMI >30 kg/m^2^ [21]. Only three respondents were categorized as underweight and were included in the normal weight group. We defined smoking into three categories: current, former and never smoking. Categories of alcohol use included: no drinking, moderate drinking (<three glasses per day) and (very) excessive drinking (≥three glasses per day). Self-reported level of education and net monthly household income were used as indicators of SEP. For level of education, respondents were asked to indicate the highest level of education that they had completed. They could choose from nine categories (no education completed to university degree). Based on the Dutch educational system and transition to the labor market in the Netherlands, we defined three levels: high (university, college, higher vocational, general secondary, and intermediate vocational education), middle (general intermediate, and lower vocational education), and low (elementary education or less). For household income, we presented several categories of income and asked respondents to indicate the category that corresponded best to their own income level. Those who indicated to have a partner income on top of their own income were asked whether they could indicate the total net income. The categories ranged from 1 (454-567 Euro per month) to 11 (2,270 Euro or more per month). We defined three levels: high (>1,816 Euro per month), middle (1,135-1,816 Euro per month) and low (<1,135 Euro per month). The middle income category covered the Dutch net modal household income of 2007 [10]. For respondents with a partner living in the same household, total household income was multiplied by 0.7 to be able to compare incomes of multi-person households to single person households [21]. The number of diet-related chronic diseases was determined during the general interview in the fourth LASA cycle (2005-2006). Respondents were asked whether they had any of the following diseases: cardiac diseases (including myocardial infarction), peripheral atherosclerosis, stroke, hypertension and diabetes mellitus (DM). To define co-morbidity we created three categories: no chronic disease; one chronic disease and two or more chronic diseases. To investigate the type of diet-related chronic disease we created three groups: cardiovascular disease (CVD, including cardiac diseases, peripheral atherosclerosis and stroke), hypertension and DM. For depressive symptoms we used the Centre for Epidemiologic Studies Depression scale, with scores ranging from 0 to 60 [22]. A score of ≥16 indicated depressive symptoms [23]. Walking disability was assessed by the question “Can you walk outside during five minutes without stopping”? We dichotomized the outcome in yes/no. Self-rated health was evaluated by the question about the perception of one’s health in general. There were five response categories ranging from (1) ‘excellent’ to (5) ‘poor’. These responses were dichotomized in ‘good’ and ‘poor’. Knowledge about healthy eating was assessed with the question “How important are the following aspects for healthy eating”, after which seven nutritional aspects were provided, including high fruit intake, high vegetable intake, high fish intake, high meat intake, high fiber intake, low salt intake and variation [24,25]. We asked respondents to indicate on a five point Likert scale if these aspects were; very unimportant to very important. For every answer 0, 1 or 2 points were assigned depending on the fact if the aspect is considered important for eating healthily. The summed score was used as an indicator of nutritional knowledge, with higher scores indicating more knowledge (Cronbach’s alpha: 0.73).

### Statistical analyses

We used descriptive statistics to summarize the characteristics of the study sample and the reported motivations to eat healthily. Logistic regression analyses were used to investigate the association between each factor and the motivations to eat healthily. We presented the univariate analyses and the multivariate analyses, where we adjusted the factors associated with the motivations for each other to study the independent effect. Odds ratios (OR) and their 95% confidence intervals were presented. In additional analyses we examined the role of specific chronic diseases as factors potentially associated with the motivations to eat healthily. In a subsample (N = 833 with available data) we investigated nutrition knowledge as a factor potentially associated with motivations to eat healthily. Tests for trend were performed by including the categorical factors as ordinal variables in the models. Interactions of the determinants with sex and with age were also tested. Data were analyzed using SAS 9.1 (SAS Inc., Cary, USA, 2004). Statistical significance was defined as a two-tailed P <0.05.

## Results

Characteristics of the study sample are presented in Table 1. The total study sample comprised 1,050 respondents, 553 females and 497 males, with a mean age of 68.9 years (SD: 6.2 years). About half of the respondents (48.1%) were overweight and 22.7% were obese. More than half of the respondents had a middle level of education (57.6%) and the majority had a middle level of income (62.9%). The most reported diet-related chronic disease was hypertension (35.5%) followed by cardiovascular diseases (27.0%).

**Table 1:** **Characteristics of the study sample (n = 1,050)**

	**N (%)**
Female	553 (52.7)
Age (years)	
< 65	337 (32.1)
65-69.9	276 (26.3)
70-74.9	219 (20.9)
≥ 75	218 (20.8)
Education	
Low	227 (21.6)
Medium	605 (57.6)
High	218 (20.8)
Household income per month (net)	
Low (<1,135 Euro)	247 (23.5)
Medium (1,135-1,816 Euro)	660 (62.9)
High (>1,816 Euro)	143 (13.6)
Weight status (BMI)	
Normal (18.5-24.9 kg/m^2^)	290 (27.6)
Overweight (25-29.9 kg/m^2^)	505 (48.1)
Obesity (≥30 kg/m^2^)	238 (22.7)
Missing	17 (1.6)
Smoking	
Never smoked	388 (37.0)
Former	487 (46.4)
Current	167 (15.9)
Missing	8 (0.8)
Alcohol use	
Non-drinker	228 (21.7)
Moderate (<3 glasses per day)	586 (55.8)
(Very) excessive (≥3 glasses per day)	236 (22.5)
Number of diet related chronic diseases	
0	516 (49.1)
1	331 (31.5)
2 or more	203 (19.3)
Presence of diet related chronic diseases specific	
CVD	283 (27.0)
Hypertension	373 (35.5)
Diabetes Mellitus	94 (9.0)
Walking disability	
Yes	100 (9.5)
Missing	2 (0.2)
Self-rated health	
Good	754 (71.8)
Poor	296 (28.2)
Depressive symptoms	
Yes	86 (8.2)
Nutritional knowledge (range: 0-14)	
Low (≤5)	273 (26.0)
Medium (6-9)	467 (44.5)
High (≥10)	141 (13.4)
Missing	169 (16.1)

*BMI* Body Mass Index, *CVD* Cardio Vascular Diseases.

The frequencies of the reported motivations to eat healthily are presented in Figure 1. The most reported motivations were “feeling fit” (51.7%, N = 543), “current health” (49.7%, N = 522), “body weight” (39.2%, N = 412) and “to prevent diseases in the future” (28.7%, N = 301). The least reported motivations were “appearance” (2.6%, N = 27), “current disease” (8.5%, N = 89) and “taste preference” (14.5%, N = 152). Most respondents (78%, N = 920) reported two motivations to eat healthily and 96 (9.1%) respondents reported a single motivation. Despite the instruction to choose a maximum of two motivations, 34 (3.3%) respondents indicated more than two motivations. Six (0.6%) respondents indicated to have other motivations to eat healthily: my vegetarian lifestyle, tradition, not important, attention to and care for food, never thought of it and avoidance of feeling guilty.

The univariate associations between the potential factors and the reported motivations to eat healthily are presented in Table 2. Females were less likely to report “feeling fit”, but more likely to report “body weight” as motivations than males. The oldest old were more likely to report “current health”, but less likely to report “to prevent diseases” as motivations than the younger old. Respondents with lower income and lower education levels were less likely to report “to prevent diseases” (OR income low vs. high: 0.40, 95% CI: 0.26-0.63, OR education low vs. high: 0.43, 95% CI: 0.28-0.64) as a motivation. The lower educated were also more likely to report “taste preference” and “current disease” as motivations. Older adults with obesity were less likely to report “current health” (OR obese vs. normal: 0.46, 95% CI: 0.33-0.66), but more likely to report “body weight” (OR obese vs. normal: 2.84, 95% CI: 1.99-4.06) as motivations than those with a normal weight. Current smokers were less likely to report “to prevent diseases” but more likely to report “current disease” as motivations compared to former and nonsmokers. Excessive and moderate alcohol drinkers were less likely to report “current disease” (OR excessive vs. nondrinker: 0.30, 95% CI: 0.16-0.58) as a motivation compared to nondrinkers. Older adults with chronic diseases were less likely to report “feeling fit” (OR two or more chronic diseases vs. no chronic diseases: 0.65, 95% CI: 0.47-0.90) than those without chronic diseases, but were more likely to report “current disease” as motivations. Those with a poor self-rated health and walking disabilities were less likely to report “feeling fit”, and both more likely to report “current disease” as motivations.

**Table 2:** **Univariate associations between reported motivations to eat healthily and the factors (OR (95% CI) in 1,050 older persons)**

	**N†**	**Feeling fit (fit) N = 543**	**N†**	**My current health N = 522**	**N†**	**Body weight N = 412**	**N†**	**To prevent disease N = 301**	**N†**	**Taste preference N = 152**	**N†**	**My current disease N = 89**	**N†**	**Appearance N = 27**
Gender														
Male	275	1.00	262	1.00	170	1.00	146	1.00	66	1.00	43	1.00	13	1.00
Female	268	0.76 (0.59-0.97)	260	0.79 (0.62-1.01)	242	1.49 (1.16-1.92)	155	0.93 (0.72-1.22)	86	1.20 (0.95-1.70)	46	0.96 (0.62-1.48)	14	0.97 (0.45-2.07)
Age (years)														
<65	171	1.00	161	1.00*	130	1.00	105	1.00	52	1.00	22	1.00	4	1.00
65-69	155	1.25 (0.91-1.72)	128	0.95 (0.69-1.31)	105	0.98 (0.71-1.36)	82	0.94 (0.66-1.33)	25	0.55 (0.33-0.91)	23	1.31 (0.71-2.40)	10	3.14 (0.97-10.12)
70-74	113	1.03 (0.73-1.45)	106	1.02 (0.73-1.44)	87	1.05 (0.74-1.48)	63	0.89 (0.61-1.29)	42	1.30 (0.83-2.03)	20	1.44 (0.76-2.70)	4	1.55 (0.38-6.25)
75+	104	0.88 (0.63-1.24)	127	1.52 (1.08-2.14)	90	1.12 (0.79-1.58)	51	0.67 (0.46-0.99)	33	0.98 (0.61-1.57)	24	1.78 (0.97-3.24)	9	3.58 (1.09-11.76)
Income														
Low	128	1.33 (0.88-2.01)	125	0.78 (0.52-1.19)	108	1.40 (0.92-2.24)	57	0.40 (0.26-0.63)	41	1.83 (0.96-3.50)	26	1.99 (0.87-4.51)	9	1.31 (0.40-4.35)
Middle	351	1.39 (0.97-2.00)	316	0.70 (0.48-1.00)	253	1.11 (0.76-1.62)	183	0.51 (0.35-0.74)	97	1.58 (0.87-2.86)	55	1.53 (0.71-3.28)	14	0.75 (0.24-2.31)
High	64	1.00	81	1.00	51	1.00	61	1.00*	14	1.00	8	1.00	4	1.00
Education														
Low	110	0.84 (0.58-1.22)	122	1.27 (0.88-1.85)	98	1.32 (0.90-1.93)	52	0.43 (0.28-0.64)	34	1.94 (1.06-3.55)	31	1.98 (1.05-3.73)	6	0.95 (0.30-2.99)
Middle	319	1.01 (0.74-1.38)	297	1.07 (0.79-1.46)	235	1.12 (0.81-1.54)	160	0.52 (0.38-0.72)	100	2.20 (1.30-3.72)	42	0.94 (0.52-1.71)	15	0.90 (0.34-2.34)
High	114	1.00	103	1.00	79	1.00	89	1.00*	18	1.00	16	1.00*	6	1.00
Weight status														
Normal	169	1.00*	153	1.00	77	1.00*	93	1.00	42	1.00	28	1.00	5	1.00
Overweight	255	0.78 (0.59-1.04)	272	0.98 (0.74-1.30)	207	1.86 (1.36-2.53)	140	0.82 (0.60-1.12)	69	0.99 (0.66-1.50)	32	0.60 (0.36-1.00)	13	1.59 (0.56-4.50)
Obesity	115	0.72 (0.52-1.02)	84	0.46 (0.33-0.66)	122	2.84 (1.99-4.06)	64	0.80 (0.55-1.16)	41	1.31 (0.82-2.10)	26	1.09 (0.63-1.90)	9	2.37 (0.79-7.18)
Smoking														
Never	199	1.00	198	1.00	161	1.00	118	1.00	57	1.00	22	1.00*	11	1.00
Former	254	1.05 (0.81-1.37)	243	0.97 (0.75-1.27)	185	0.86 (0.66-1.13)	144	0.96 (0.72-1.28)	67	0.91 (0.63-1.33)	45	1.66 (0.98-2.79)	13	0.96 (0.43-2.17)
Current	87	1.06 (0.74-1.52)	78	0.86 (0.60-1.23)	62	0.83 (0.58-1.21)	36	0.63 (0.41-0.96)	26	1.06 (0.64-1.75)	21	2.35 (1.26-4.37)	3	1.64 (0.18-2.34)
Alcohol use														
Non drinker	110	1.00	115	1.00	89	1.00	63	1.00	30	1.00	37	1.00*	8	1.00
Moderate	316	1.26 (0.93-1.71)	291	0.98 (0.72-1.32)	229	1.01 (0.74-1.38)	166	1.04 (0.74-1.46)	90	1.20 (0.77-1.87)	39	0.37 (0.23-0.60)	7	0.82 (0.35-1.94)
Excessive	117	1.06 (0.73-1.52)	116	0.95 (0.66-1.37)	94	1.03 (0.71-1.50)	72	1.15 (0.77-1.72)	32	1.04 (0.61-1.77)	13	0.30 (0.16-0.58)	2	0.24 (0.05-1.12)
Chronic diseases**														
0	293	1.00*	259	1.00	193	1.00	150	1.00	68	1.00	20	1.00*	4	1.00
1	157	0.69 (0.52-0.91)	163	0.96 (0.73-1.27)	130	1.08 (0.81-1.44)	105	1.14 (0.84-1.53)	61	1.49 (1.02-2.17)	23	1.85 (0.73-2.23)	15	1.28 (0.53-3.13)
2 or more	93	0.65 (0.47-0.90)	100	0.97 (0.70-1.34)	89	1.32 (0.95-1.83)	46	0.72 (0.49-1.05)	23	0.85 (0.51-1.40)	46	7.30 (4.19-12.71)	5	1.65 (0.63-4.31)
Self-rated health														
Good	412	1.00	381	1.00	291	1.00	225	1.00	105	1.00	54	1.00	9	1.00
Poor	131	0.65 (0.50-0.86)	141	0.88 (0.67-1.15)	121	1.09 (0.83-1.43)	76	0.81 (0.60-1.09)	47	1.16 (0.80-1.68)	35	2.55 (2.91-7.14)	18	1.28 (0.57-2.87)
Walking disability														
No	503	1.00	472	1.00	370	1.00	276	1.00	139	1.00	66	1.00	22	1.00
Yes	38	0.55 (0.36-0.83)	49	0.98 (0.65-1.47)	41	1.09 (0.72-1.66)	25	0.82 (0.51-1.31)	13	0.87 (0.47-1.61)	22	3.78 (2.22-6.46)	5	2.22 (0.82-6.01)
Depressive symptoms														
No	501	1.00	482	1.00	376	1.00	277	1.00	142	1.00	72	1.00	25	1.00
Yes	42	0.89 (0.57-1.38)	40	0.88 (0.56-1.36)	36	1.13 (0.72-1.77)	24	0.96 (0.59-1.58)	10	0.77 (0.39-1.51)	17	3.06 (0.71-5.48)	2	0.90 (0.21-3.85)

*Statistically significant trend across categories (P value <0.05).

**Number of diet related chronic diseases.

†Number of respondents that reported this motivation to eat healthily.

To determine which variables were independently associated with the motivations to eat healthily, we performed multivariate analyses adjusting the factors for each other (Table 3). After adjustment for BMI and the other factors, females were still less likely to report “feeling fit”, but more likely to report “body weight” as motivations. The oldest old were more likely to report “current health” than the younger old, even though this association was adjusted for diet-related chronic diseases and the other factors. The SEP indicators were also adjusted for each other and older adults with lower incomes were still less likely to report “to prevent diseases” (OR low income vs. high: 0.52, 95% CI: 0.32-0.86), but more likely to report “feeling fit” (OR low vs. high: 1.97, 95% CI: 1.13-2.86) as motivations than those with higher incomes. The lower educated were also less likely to report “to prevent diseases” (OR low vs. high: 0.43, 95% CI: 0.27-0.70), but more likely to report “current health” (OR low vs. high: 1.69, 95% CI: 1.09-2.60) as motivations than the higher educated. Those with overweight and obesity were less likely to report “feeling fit”, but more likely to report “body weight” (OR obese vs. normal: 2.78, 95% CI: 1.88-4.11) as motivations than those with normal weight. Older adults with obesity were also less likely to report “current health” (OR obese vs. normal: 0.47, 95% CI: 0.32-0.69) as a motivation. Excessive and moderate alcohol users were less likely to report “current disease” (OR excessive vs. nondrinker: 0.37, 95% CI: 0.17-0.83) as a motivation than the nondrinkers. Those with chronic diseases were more likely to report “current disease” (OR two or more vs. no chronic disease: 4.41, 95% CI: 2.31-8.44) as a motivation than those without chronic disease. Those with a poor self-rated health were more likely to report “current disease” (OR poor vs. good: 2.31, 95% CI: 1.22-3.73) as a motivation than those with a good self-rated health. We observed no interactions between any of the factors and sex and age (P > 0.05) in the multivariate model.

**Table 3:** **Multivariate associations between reported motivations to eat healthily and the factors, adjusted for each other (OR (95% CI) in 1,050 older persons)**

	**N†**	**Feeling fit (fit) N = 543**	**N†**	**Current health N = 522**	**N†**	**Body weight N = 412**	**N†**	**To prevent disease N = 301**	**N†**	**Taste preference N = 152**	**N†**	**Current disease N = 89**	**N†**	**Appearance N = 27**
Gender														
Male	275	1.00	262	1.00	170	1.00	146	1.00	66	1.00	43	1.00	13	1.00
Female	268	0.74 (0.56-0.97)	260	0.76 (0.58-1.01)	242	1.52 (1.14-2.01)	155	1.00 (0.74-1.36)	86	1.13 (0.77-1.68)	46	0.75 (0.44-1.29)	14	0.85 (0.36-2.02)
Age (years)														
<65	171	1.00	161	1.00*	130	1.00	105	1.00	52	1.00	22	1.00	4	1.00
65-69	155	1.31 (0.94-1.82)	128	1.00 (0.72-1.40)	105	0.90 (0.64-1.27)	82	0.99 (0.74-1.36)	25	0.54 (0.32-0.91)	23	0.95 (0.47-1.59)	10	2.87 (0.87-9.43)
70-74	113	1.18 (0.81-1.72)	106	1.13 (0.77-1.65)	87	0.88 (0.60-1.31)	63	0.99 (0.69-1.42)	42	1.17 (0.71-1.93)	20	0.74 (0.34-1.51)	4	1.40 (0.32-6.07)
75+	104	1.07 (0.72-1.60)	127	1.81 (1.20-2.72)	90	0.98 (0.65-1.48)	51	0.73 (0.66-1.50)	33	0.78 (0.44-1.37)	24	0.70 (0.33-1.01)	9	2.95 (0.78-11.17)
Income														
Low	128	1.79 (1.13-2.86)	125	0.65 (0.41-1.04)	108	1.33 (0.82-2.15)	57	0.52 (0.32-0.86)	41	1.57 (0.77-3.18)	26	0.96 (0.37-2.45)	9	0.96 (0.24-3.89)
Middle	351	1.58 (1.08-2.32)	316	0.61 (0.41-0.90)	253	1.10 (0.73-1.63)	183	0.57 (0.39-0.85)	97	1.45 (0.78-2.69)	55	1.12 (0.49-2.58)	14	0.67 (0.20-2.21)
High	64	1.00*	81	1.00	51	1.00	61	1.00*	14	1.00	8	1.00	4	1.00
Education														
Low	110	1.04 (0.68-1.60)	122	1.69 (1.09-2.60)	98	1.00 (0.64-1.55)	52	0.43 (0.27-0.70)	34	1.55 (0.79-3.04)	31	1.09 (0.49-2.40)	6	0.67 (0.18-2.54)
Middle	319	1.06 (0.80-1.56)	297	1.32 (0.95-1.85)	235	0.89 (0.63-1.25)	160	0.55 (0.39-0.78)	100	1.86 (1.08-3.24)	42	0.69 (0.35-1.37)	15	0.71 (0.14-2.03)
High	114	1.00	103	1.00*	79	1.00	89	1.00*	18	1.00	16	1.00	6	1.00
Weight status														
Normal	169	1.00*	153	1.00	77	1.00*	93	1.00	42	1.00	28	1.00	5	1.00
Overweight	255	0.74 (0.55-1.00)	272	1.00 (0.74-1.34)	207	1.98 (1.43-2.73)	140	0.86 (0.62-1.19)	69	0.85 (0.55-1.31)	32	0.56 (0.31-1.01)	13	1.58 (0.54-4.57)
Obesity	115	0.84 (0.58-1.22)	84	0.47 (0.32-0.69)	122	2.78 (1.88-4.11)	64	0.86 (0.57-1.30)	41	1.17 (0.70-1.97)	26	0.52 (0.26-1.03)	9	1.89 (0.57-6.25)
Smoking														
Never	199	1.00	198	1.00	161	1.00	118	1.00	57	1.00	22	1.00	11	1.00
Former	254	1.03 (0.76-1.38)	243	0.97 (0.72-1.31)	185	0.89 (0.65-1.21)	144	0.99 (0.72-1.37)	67	0.82 (0.53-1.25)	45	1.65 (0.88-3.09)	13	1.16 (0.46-2.92)
Current	87	1.02 (0.69-1.50)	78	0.79 (0.53-1.16)	62	0.86 (0.57-1.28)	36	0.67 (0.43-1.06)	26	1.10 (0.64-1.88)	21	1.87 (0.90-3.88)	3	0.86 (0.22-3.29)
Alcohol use														
Non drinker	110	1.00	115	1.00	89	1.00	63	1.00	30	1.00	37	1.00*	8	1.00
Moderate	316	1.07 (0.75-1.51)	291	1.00 (0.70-1.42)	229	1.27 (0.89-1.82)	166	0.76 (0.52-1.12)	90	1.41 (0.86-2.33)	39	0.55 (0.30-1.00)	7	0.83 (0.31-2.22)
Excessive	117	0.88 (0.58-1.33)	116	0.96 (0.63-1.46)	94	1.40 (0.91-2.16)	72	0.81 (0.51-1.28)	32	1.32 (0.72-2.43)	13	0.37 (0.17-0.83)	2	0.23 (0.05-1.22)
Chronic diseases**														
0	293	1.00	259	1.00	193	1.00	150	1.00	68	1.00	20	1.00*	4	1.00
1	157	0.73 (0.55-0.98)	163	0.97 (0.72-1.30)	130	1.04 (0.77-1.41)	105	1.16 (0.84-1.60)	61	1.46 (0.98-2.20)	23	1.53 (0.78-2.99)	15	1.00 (0.38-2.61)
2 or more	93	0.75 (0.52-1.10)	100	1.00 (0.68-1.47)	89	1.26 (0.85-1.85)	46	0.75 (0.49-1.17)	23	0.78 (0.44-1.40)	46	4.41 (2.31-8.44)	5	1.31 (0.44-3.88)
Self-rated health														
Good	412	1.00	381	1.00	291	1.00	225	1.00	105	1.00	54	1.00	9	1.00
Poor	131	0.74 (0.54-1.02)	141	0.92 (0.66-1.27)	121	0.90 (0.65-1.26)	76	1.02 (0.71-1.47)	47	1.27 (0.82-1.98)	35	2.13 (1.22-3.73)	18	0.84 (0.32-2.22)
Walking disability														
No	503	1.00	472	1.00	370	1.00	276	1.00	139	1.00	66	1.00	22	1.00
Yes	38	0.78 (0.47-1.29)	49	1.16 (0.70-1.94)	41	1.76 (0.45-1.29)	25	1.02 (0.58-1.82)	13	0.54 (0.25-1.16)	22	1.64 (0.80-3.36)	5	1.76 (0.50-6.20)
Depressive symptoms														
No	501	1.00	482	1.00	376	1.00	277	1.00	142	1.00	72	1.00	25	1.00
Yes	42	1.01 (0.62-1.66)	40	0.87 (0.53-1.45)	36	1.12 (0.67-1.86)	24	1.13 (0.66-1.96)	10	0.74 (0.35-1.57)	17	1.18 (0.54-2.58)	2	0.92 (0.20-4.29)

*Statistically significant trend across categories (P value <0.05).

**Number of diet related chronic diseases.

†Number of respondents that reported this motivation to eat healthily.

In additional multivariate analyses we investigated the role of specific diet related chronic diseases (CVD, hypertension and DM) and nutritional knowledge in motivations to eat healthily. Older adults with CVD were less likely to report “to prevent diseases” (OR: 0.68, 95% CI: 0.48-0.97), but were more likely to report “current disease” (OR: 2.59, 95% CI: 1.54-4.33) as motivations than those without CVD. Those with hypertension were also more likely to report “current disease” (OR: 1.67, 95% CI: 1.03-2.72) as a motivation than those without hypertension. Older adults with DM were less likely to report “feeling fit” (OR: 0.53, 95% CI: 0.34-0.84), but more likely to report “current disease” (OR: 3.85, 95% CI: 2.12-6.99) as motivations than those without DM. Older adults with a higher nutritional knowledge score (≥10 points) were more likely to report “to prevent diseases” (OR: 2.17, 95% CI: 1.37-3.45) as a motivation than those with a lower score (≤5 points). We observed no other associations between nutritional knowledge and motivations to eat healthily.

## Discussion

In this study we aimed to identify what motivations Dutch older adults have to eat healthily in a large population-based sample and to investigate if these motivations were associated with SEP and other demographic, lifestyle and health characteristics. We showed that the top three most reported motivations were: “feeling fit”, “current health” and “body weight”. More importantly, the reported motivations did largely depend on the characteristics of the older adults. Older adults with physical health problems and a poor self-rated health were more likely to report “current disease” as a motivation to eat healthily. Older adults with lower SEP were less likely to report “to prevent diseases” and those with obesity were less likely to report “current health” as motivations to eat healthily.

In our study “current health” and “feeling fit” were the most reported motivations for the overall sample. This is consistent with other studies that showed that health status is an important motivation to eat healthily in adults [11,15,26] but also important in older adults [14,27] This is not surprising, because older adults are most likely to suffer from diseases and their consequences. It is not clear from the literature whether having health related motivations to eat healthily is more strongly associated with a healthy diet than other motivations. Previous research did show that health related motivations are important determinants for a healthy diet [28,29]. However, Satia et al. showed that health related motivations were not associated with dietary change after a 12 month randomized intervention trial among adults [26]. It should be noted that the study was performed in a relatively healthy sample, and health concerns therefore may have played a minor role. In our study “current disease” was especially important for those with chronic diseases and for those with a poor self-rated health. This may indicate that those with chronic diseases and a poor self-rated health are aware of the link between nutrition and health and that the presence of a disease is an important motivation to eat healthily in older adults.

Special attention should be paid to lower SEP groups. Those with lower incomes and lower education levels were less likely to report “to prevent diseases” as a motivation to eat healthily, even after adjusting the SEP indicators for each other and the other factors. A study among US older adults also found that those with lower education levels were less likely to report preventing disease as a motivation to eat healthily [14]. A study among adults showed that a lower income was related to less importance of health considerations in food selection [12]. These results are remarkable because it is known that low SEP is associated with a higher risk of diet-related chronic diseases [30]. One explanation could be that higher levels of education provide greater access to health information, knowledge and cognitive benefits that are preventive for diet-related chronic diseases [31]. Indeed, lower education levels are associated with a lower nutrition knowledge including the diet-disease links [32] and this knowledge was found to be a mediator in the SEP variation in dietary intake, especially for vegetables and fruit [33]. A second explanation could be that our findings reflect the fact that older adults with higher incomes have more financial freedom to take health aspects into account in their food choice, because of the higher costs of healthy foods [34]. This is confirmed by others who showed that adults with lower incomes and education levels place more importance on price than on health in their food selection [35]. These findings might indicate that in low SEP groups more immediate problems, such as a shortage of money to buy healthy foods are barriers for focusing on long term issues related to food, such as the prevention of diseases in the future.

Older adults with overweight and obesity were more likely to report “body weight” as a motivation to eat healthily than those with normal weight. This is consistent with other studies that showed that body weight or reducing body weight are important motivations to eat healthily [19,26]. However, older adults with obesity were less likely to report “current health” as a motivation to eat healthily. Furthermore, the motivation “to prevent diseases” was not associated with weight status. From a public health perspective this is surprising, because the relationship between body weight and health status is well established [36]. From a well-being perspective this might be less surprising, because obesity is a complex and multifactorial disease. It also impacts physical function, self-esteem and quality of life [37,38], which may provide additional motivations to eat healthily for people with obesity, compared to the prospect of improving future health only. This might reflect that the presence of overweight or obesity and their direct consequences are more important motivations to eat healthily than possible future health consequences.

In the overall sample, the prevention of disease was chosen by only 28.5% of the older adults, while almost half (49.2%) of the older adults were free from diet-related chronic diseases and 71.1% rated their self-perceived health as excellent or good. One might expect that prevention of disease as a motivation to eat healthily would be of more importance in older age as the risk of chronic diseases in old age is relatively high and a healthy diet can prevent diet-related chronic diseases [39-42]. Healthy eating is not only of importance in younger aged; also in older aged it is associated with increased life expectancy [5,6]. In additional analyses (data not shown) we found that older adults with a higher nutritional knowledge score were more likely to report “to prevent diseases” as a motivation than those with a lower score. This might indicate that some older adults remain unaware of the link between healthy eating and disease prevention.

The results of our study should be considered in the context of its strengths and limitations. This study is one of the first that presents underlying motivations to eat healthily in the old aged and more importantly showed that the reported motivations largely depend on the characteristics of older adults. This adds information to the existing literature and provides further insight in why older adults eat what they eat. Furthermore, the LASA study is a well-characterized study with a high response rate. This enabled us to study a large sample of community dwelling older Dutch adults. Respondents were selected on having a normal cognitive status, which has the important advantage that recall bias due to poor cognitive functioning with regard to self-reported data is limited. Moreover, only six of the 1,050 respondents reported other motivations to eat healthily than the ones presented in the questionnaire, which suggests that the most important motivations to eat healthily were included in our study. Furthermore, to study the independent associations between the factors and motivations, we adjusted the factors in the multivariate analyses for each other. However, this does not justify the underlying causal structure of all factors in their association with eating healthily via complex relations of confounding and mediation. However, it should be noted that our goal was not to test complex causal structures, but to identify subgroups and their particular sets of motivations to eat a healthy diet. Furthermore, we asked respondents to indicate their most important motivation to eat healthily. This answer might depend on the interpretation of the concept of healthy eating by the older adult in question. A final limitations is the cross-sectional design of this study and any causal associations can only be inferred.

The findings of this study suggest that promotion messages to eat a healthy diet should differ between subgroups of older adults. The message to older adults with a disease or poor self-rated health needs to focus on the presence of disease or health problem and the link with eating healthily, because this appears to be an important reason for them to eat a healthy diet. For low SEP and obese older adults disease prevention seem to be a less important motivation to eat healthily, even though a healthy diet could improve their health. Promotion messages aiming at disease prevention and eating healthily might therefore be less effective in these groups. Information and coaching about weight status and the ways a healthy diet contributes to weight loss may be more effective for the obese group. Future research should find out why low SEP groups find disease prevention of less importance. Do these older adults lack necessary knowledge about the link between nutrition and disease or is the low SEP and its associated problems a bigger issue than eating a healthy diet? Before developing tailored interventions based on motivations to eat healthily, a complete understanding of the pathway between the motivations and characteristics of the older adults is necessary.

## Conclusions

In conclusion, the results of our study suggest that “feeling fit”, “current health” and “body weight” were the most important motivations to eat healthily in a large sample of older Dutch adults. Multivariate analyses showed that the importance of the reported motivations largely depended on the characteristics of the older adults. The presence of a disease seems to be an important motivation to eat healthily, which indicates that older adults with self-perceived or reported health problems are aware of the link between their disease and nutrition. Special attention should be paid towards lower SEP groups and obese older adults, because disease prevention seems to be a less important motivation for these older adults.
